# Syndrome du défilé thoraco-brachial neurologique chez l’enfant: à propos d’un cas

**DOI:** 10.11604/pamj.2018.30.296.14523

**Published:** 2018-08-30

**Authors:** Ghizlan El Amri, Mohammed Zalagh, Ali Jahidi, Fouad Benariba

**Affiliations:** 1 Service d’ORL et de Chirurgie Cervico-faciale, Hôpital Militaire d’Instruction Mohamed V, Faculté de Médecine, Université Mohamed V, Rabat, Maroc

**Keywords:** Côte cervicale surnuméraire, défilé thoraco-abdominal, enfant, Supernumerary cervical rib, thoracic outlet syndrome, child

## Abstract

Le Syndrome du Défilé Thoraco-brachial (SDTB) correspond à l’ensemble des manifestations cliniques liées à la compression des troncs du plexus brachial et ou des vaisseaux sous-claviers lors de la traversée cervico-thoraco-brachiale. Les formes pédiatriques sont rares. Les auteurs rapportent le cas d’une jeune fille traitée pour SDTB neurologique, conséquence d’une côte cervicale surnuméraire.

## Abstract (en)

Thoracic Outlet Syndrome (NTOS) encloses the whole set of clinical manifestations associated with the compression of the trunks of the brachial plexus and/or of the subclavian vessels as they cross the thoracic outlet (cervico-thoraco-brachial junction). It rarely affects children. We here report the case of a young girl treated for neurologic NTOS caused by supernumerary cervical rib.

## Introduction

Le syndrome du défilé thoraco-brachial (SDTB) correspond à l’ensemble des manifestations cliniques liées à la compression des troncs du plexus brachial et ou des vaisseaux sous- claviers lors de la traversée cervico-thoraco-brachiale. Les formes neurologiques sont de loin les plus fréquentes, regroupant environ 95% des cas chez l’adulte. Les formes pédiatriques sont rares [1]. Nous rapportons le cas d’une jeune fille traitée pour SDTB neurologique, conséquence d’une côte cervicale surnuméraire.

## Patient et observation

**Figure 1:**
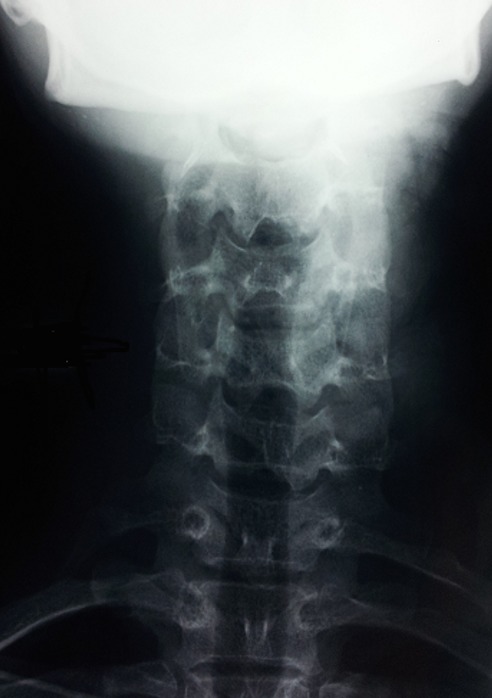
Radiographie standard de face

**Figure 2:**
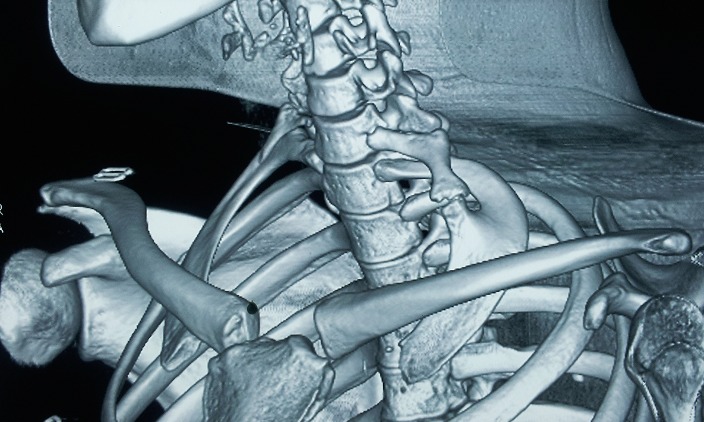
Scanner 3D

Une jeune fille de 14 ans nous a été adressée pour une tuméfaction sus-claviculaire gauche évoluant depuis un an. L’enfant décrivait des douleurs à type de paresthésies, particulièrement de la main. Ces paresthésies sont aggravées par l’effort et le décubitus latéral gauche. L’examen clinique retrouve une tuméfaction dure, sensible et non pulsatile, mesurant 3cm, au niveau du creux sus-claviculaire gauche. Les pouls étaient présents. Aucun déficit moteur ou sensitif n’a été noté. Les manoeuvres provocatrices étaient négatives. Il n’a pas été noté d’amyotrophie des éminences thénar et hypothénar. Les radiographies du rachis cervical de face (Figure 1) et le scanner (Figure 2) avec reconstruction tridimensionnelle ont permis la mise en évidence de deux côtes surnuméraires cervicales droite et gauche.

La côte cervicale gauche présente une zone de synchondrose avec la première côte thoracique. L’électromyographie n’a pas objectivé de déficit. Un echodoppler a montré un flux normal dans les vaisseaux sous claviers. Une résection de cette côte gauche surnuméraire et de la première côte homolatérale était réalisée par voie sus-claviculaire. Dés le lendemain de l’intervention, les paresthésies du membre supérieur gauche ainsi que la tuméfaction sus-claviculaire avaient disparu. À un an de recul l’enfant est parfaitement asymptomatique.

## Discussion

Le SDTB est un ensemble de signes résultant de la compression des structures neuro-vasculaires lors de la traversée thoraco-brachiale. La forme neurologique par compression du plexus brachial est la plus fréquente, représentant 95%. Les formes vasculaires veineuse ou artérielle représentent 3% et 1% respectivement [2]. Une étude de 200 patients opérés pour SDTB retrouve que 8.5% des cas étaient attribués à une côte cervicale surnuméraire [3]. La majorité des cas (43%) étaient dus à des variations d’insertions des muscles scalènes et sous claviers et 10% avaient un muscle scalène surnuméraire. La pluparts des formes acquises des SDTB sont d’origine inflammatoire post-traumatique [4]. Un SDTB neurologique résultant d’une anomalie de côte cervicale révélé par une tuméfaction sus-claviculaire chez un enfant est une condition très rare. Chez cette population de jeunes adolescents les manifestations vasculaires de STDB atteint les 56% alors qu’elles ne dépassent pas les 10% chez l’adulte [5]. Cette disparité pourrait s’expliquer par le fait que les SDTB neurologiques résultent souvent d'un traumatisme d'hyperextension (lésion du coup de fouet cervical) ou d'une activité physique élevée (surutilisation) chez des patients présentant des anomalies congénitales telles que des côtes cervicales, des bandes congénitales ou fusion de côtes.

Le diagnostic d’un SDTB neurologique est clinique. Parmi les signes les plus fréquents les paresthésies dans le territoire C8-T1 (lower tract syndrome) des anglo-saxons. La douleur de la région sus-claviculaire et les céphalées sont souvent associées a des anomalies osseuses: méga-apophyse, côte cervicale, ou tractus fibreux (upper tract syndrome). L’inspection et la palpation de la région sus-claviculaire permettent de rechercher une masse et son caractère pulsatile. Dans notre cas la masse cervicale était le principal motif de consultation. Dans la serie de Chang *et al.* deux des cinq patients avec un SDTB neurologique présentaient une masse cervicale indolore et pulsatile [6]. L’examen de la main apprécie la trophicité des muscles intrinsèques: une hypotrophie de l éminence thénar avec déformation de la main en griffe est évocatrice d’un SDTB neurologique. Les tests de provocation Adson’s, Wrights et le test de Roos ou EAST test, sont incontournables au diagnostic en cas de reproductibilité des résultats. L’imagerie (radiographies standard et scanner) permettent de mettre en évidence les anomalies osseuses. Les tests électro-physiologiques sont utiles au diagnostic lorsqu’ils sont réalisés le bras en élévation et trouvent leur indication en cas de suspicion de dénervation des muscles intrinsèques de la main [7].

Le traitement conservateur par les exercices de rééducation, d'étirement et de renforcement posturaux n’est efficace que dans les cas diagnostiqués précocement avec symptômes mineurs. Notre patient avait déjà été traité par physio-kinésithérapie pendant 3 mois sans amélioration. Il n y a pas de consensus sur le choix de la voie d’abord chirurgicale, elle peut être axillaire, sus claviculaire ou combinée selon le type de la lésion et son étiologie. La voie axillaire de Roos (1965), semble être la meilleure technique pour la décompression de la veine et la résection du segment antérieur de la côte. La voie supra-claviculaire est beaucoup plus facile et permet de traiter les anomalies anatomiques ainsi que les lésions artérielles; la résection des côtes est limitée au segment postérieur et moyen. La première côte peut être complètement réséquée par une double approche supra / infra-claviculaire. Cette approche est également conseillée dans les cas de signes neurologiques de compression sur les racines C5-C6-C7 [8]. La scalenotomie simple sans résection de la première côte n’a pas d’intérêt. La résection de la côte cervicale et de la première côte est nécessaire pour soulager les symptômes et prévenir une récidive. Le SDTB neurologique associe des signes complexes avec souvent une douleur chronique ce qui met le chirurgien devant un challenge thérapeutique avec résultat parfait difficile à atteindre. En effet les symptômes sensoriels à type d’engourdissement et de paresthésies sont les plus souvent améliorées par la chirurgie que la douleur et les déficits moteurs [5, 8-13]. Aucun facteur prédictif de résultat chirurgical ne s’est révélé significatif, y compris l'âge, le sexe et les manœuvres provocatrices. Malgré les symptômes résiduels dans la plupart des malades opérés (67%) [9, 13]. La majorité des interventions chirurgicales ont été jugées réussies par les patients et l’entourage. La qualité de vie des patients est améliorée par l’amélioration de la mobilité des membres.

## Conclusion

Chez l’adolescent la forme vasculaire du SDTB prédomine, le SDTB neurologique reste un diagnostic d’élimination. La tuméfaction cervicale pourrait être le signe d’appel majeur. Le traitement conservateur constitue l’essentiel du traitement. La chirurgie est nécessaire dans certains cas et ses résultats sont variables.

## Conflits d’intérêts

Les auteurs ne déclarent aucun conflit d'intérêts.
